# Population Structure and Genetic Diversity of Tumbler Pigeon Based on Whole-Genome Resequencing

**DOI:** 10.3390/ani16152343

**Published:** 2026-08-01

**Authors:** Xiaomei Ma, Haiying Li, Xiangxi Liu, Xiaobin Li, Hao Zhang, Yingping Wu, Xiaoyu Zhao

**Affiliations:** 1 College of Animal Science, Xinjiang Agricultural University, Urumqi 830091, China; 17389410013@163.com (X.M.); lhy-3@163.com (H.L.); 17799623750@163.com (X.L.); lxb262819@163.com (X.L.); wyp_941208@163.com (Y.W.); 2 Key Laboratory of Animal Nutrition and Feed, Yili Vocational and Technical College, Yining 835000, China; 13579729168@163.com

**Keywords:** Tumbler pigeon, whole-genome resequencing, genetic diversity, genetic structure

## Abstract

The Tumbler pigeon, a genetic resource of pigeons, exhibits diverse feather colors and distinct flight characteristics compared with other pigeon species, but the genetic basis of these traits remains unclear. Using whole-genome resequencing technology with Illumina NovaSeq X Plus paired-end sequencing (PE150, read length 150 bp) at an average genome coverage of 14.5×, this study analyzed the genetic structure, genetic diversity, and genetic differentiation of 80 individual Tumbler pigeons, Tarim pigeons and homing pigeons, collected from different regions of Xinjiang. In principal component analysis, the Tarim pigeon (TP) and the homing pigeon (XY) each formed relatively independent clusters, and there is a detectable genetic distance between them and the Tumbler pigeon (FC, FY) from the two regions. Moreover, there is an individual overlap phenomenon between the two Tumbler pigeon populations. In the analysis of genetic diversity, *N*_a_ ranged from 1.93 to 1.97 and *N*_e_ from 1.55 to 1.56 across populations, with the TP population exhibiting the highest *N*_a_. PIC values ranged between 0.251 and 0.256, and *H*_E_ values were lower than *H*_O_ values in all four pigeon populations, indicating that moderate levels of genetic polymorphism were retained. In phylogenetic tree and population structure analyses, TP and XY formed relatively independent clusters. At the same time, individuals from the two Tumbler pigeon regions showed intermixing, resulting in the most distinct clustering pattern. Based on these findings, detectable genetic differentiation exists among the Tumbler pigeon, Tarim pigeon, and homing pigeon, suggesting that Tumbler pigeons may represent a locally differentiated genetic resource. The findings of this study will enhance our understanding of the population genetic structure of Tumbler pigeon resources, which is of great significance for promoting the conservation of Tumbler pigeon genetic resources and facilitating breed innovation and genetic improvement.

## Simple Summary

Tumbler pigeons may represent a locally differentiated genetic resource, but their genetic basis and population structure remain unclear, requiring further sampling and functional analysis. This study conducted whole-genome resequencing of 80 individuals, including Tumbler pigeons from two regions, Tarim pigeons, and homing pigeons, to investigate their genetic diversity, population structure, and differentiation patterns. Principal component analysis and phylogenetic analysis revealed that the Tarim pigeon and homing pigeon formed distinct monophyletic clades, with detectable genetic distance from Tumbler pigeon. In contrast, populations from different regions showed overlap. Population genetic analyses indicated that all four groups maintained moderate levels of genetic polymorphism, with Tarim pigeons displaying higher allelic diversity. These results suggest low genetic differentiation between Tumbler pigeons and other pigeon populations, potentially representing a locally differentiated genetic resource, although further sampling and functional analyses are needed for confirmation. This study provides critical data support for the conservation, genetic improvement, and breed innovation of Tumbler pigeons.

## 1. Introduction

The domestic pigeon (*Columba livia*) is one of the world’s oldest domesticated bird species, with a history of domestication dating back at least 5000 years, originating from the rock dove in the Middle East and Mediterranean regions [1]. In “*On the Origin of Species*”, Darwin noted that the domestic pigeon evolved from rock doves, using them as an example to illustrate the significant role of artificial selection in breed differentiation [2]. Through prolonged directional selection, rock doves have diversified into over 350 breeds exhibiting strikingly different phenotypes and behavioral traits [3]. Under precise human breeding, domestic pigeon breeds display remarkable phenotypic and behavioral diversity. The homing pigeon—also known as the racing pigeon or competitive pigeon—is a specialized breed used for communication and long-distance racing. It excels at homing, with exceptional memory and strong spatial orientation, enabling outstanding navigation and return capabilities over vast distances. Its domestication and utilization date back thousands of years, with historical records from ancient Egypt documenting its use for message delivery. After centuries of selective breeding, modern homing pigeons have become highly specialized in navigation mechanisms and flight performance. Their navigation relies on multiple cues including geomagnetic sensing, solar positioning, and visual landmarks. Notably, their hippocampus is exceptionally well-developed, giving them spatial memory capabilities far surpassing those of most other birds [4]. The Tarim pigeon, also known as Xinhe pigeon or Yarkand pigeon, is native to the Tarim Basin in the Xinjiang Uygur Autonomous Region, and is primarily raised in the Kashgar and Aksu regions [5]. This local breed is a dual-purpose pigeon, valued for both meat and eggs, characterized by strong adaptability, high reproductive capacity, good disease resistance, tolerance to coarse feed, strong foraging ability, and distinctive meat flavor. In recent years, the Tarim pigeon farming industry has developed rapidly in southern Xinjiang, where the only national-level breed conservation farm has been established to preserve and selectively breed this breed.

The Tumbler pigeon, although belonging to the same category of racing pigeon as homing pigeon, exhibits distinctly different behavioral characteristics. Tumbler pigeons possess a hereditary trait that enables them to perform aerial somersaults during flight—repeated backward or rolling flips, either individually or in flocks [6]. In “*Variation of Animals and Plants under Domestication*”, Darwin described this rolling behavior as “one of the most remarkable inherited habits on record” [7]. Subsequent research has confirmed that this behavior is not caused by epilepsy or neurological defects, but rather represents a heritable motor phenotype. As a national-level sport, the racing pigeon population has experienced rapid growth in China over recent decades, making the country the fastest-growing market for the global pigeon racing industry, with membership numbers and the number of competitions ranking first worldwide.

Xinjiang is the core distribution area of the Tumbler pigeon’s germplasm in China. Previous investigations have found that the Xinjiang Tumbler pigeon population exhibits diverse plumage colors and distinct flight characteristics compared to local pigeon breeds. However, there is currently a severe lack of production data on Tumbler pigeons, and no significant progress has been made in breed identification. There are few reports on the genetic diversity and systematic evolution of Tumbler pigeons in various regions.

With the advent of the high-throughput detection era, whole-genome resequencing (WGS) technology has become one of the key methods for in-depth study of population genetic structure [8,9]. This technology, through whole-genome variation analysis, can comprehensively assess population genetic differences and genetic diversity [10], overcoming the limited coverage of traditional molecular markers and systematically analyzing population genetic characteristics and population structure.

In recent years, the rapid development of genomic technologies has provided a new means to analyze genetic differentiation and phenotypic diversity among domestic pigeon breeds. Shapiro et al. [11] completed the assembly of the domestic pigeon reference genome, identifying over 25 million single-nucleotide polymorphism (SNP) sites and laying a crucial foundation for pigeon population genetics research. Stringham et al. [12] used microsatellite and mitochondrial DNA markers to reveal significant genetic structure differentiation among global pigeon breeds, confirming restricted gene flow and independent evolutionary trajectories among breeds. Domyan et al. [13] further clarified the genetic regulatory mechanism of pigeon plumage color variation, revealing the complex network of major gene interactions underlying phenotypic traits.

Although significant progress has been made in the genetic research of domestic pigeon, most of the existing work has focused on commercial meat pigeon breeds. The genetic analysis of the whole genome of the Xinjiang Tumbler pigeon, a unique local breed in Xinjiang, China, especially with its distinctive somersaulting flight behavior, remains unexplored. Although the Xinjiang Tumbler pigeon, the homing pigeon, and the Tarim pigeon all belong to the domestic pigeon species, long-term geographic isolation and differing breeding objectives (ornamental, racing, and meat production) may have led to the accumulation of distinguishable genetic differentiation among these populations. The Tarim pigeon and Tumbler pigeon both belong to the native domestic pigeon genetic resources of Xinjiang; the former is a locally bred meat breed listed in the national catalog, while the latter is a distinctive ornamental strain formed through long-term selective breeding, though its genetic background remains unclear. In this study, the homing pigeon, as a non-native, introduced breed, serves as an outgroup control. By comparing these three groups of pigeon, which differ in origin and breeding objectives, it is possible to assess the genetic distinctiveness of the Tumbler pigeon and its potential value as an independent genetic resource. However, no study has systematically compared the genetic diversity, population structure, and phylogenetic relationships of these three pigeons groups at the whole-genome level. This study hypothesizes that long-term geographical isolation and artificial selection have led to detectable genetic differentiation among the Xinjiang Tumbler pigeon, homing pigeon, and Tarim pigeon populations. Moreover, as an ornamental pigeon with unique movement behavior, the Xinjiang Tumbler pigeon may exhibit genetic distinctiveness from the other two groups. To verify this hypothesis, this study, based on whole-genome resequencing data, aims to clarify the population genetic structure and phylogenetic status of the three groups, assess the degree of genetic differentiation among them, and provide data support for the identification, protection, and innovative utilization of Xinjiang Tumbler pigeon germplasm resources.

## 2. Materials and Methods

### 2.1. Sample Collection

**Figure 1:**
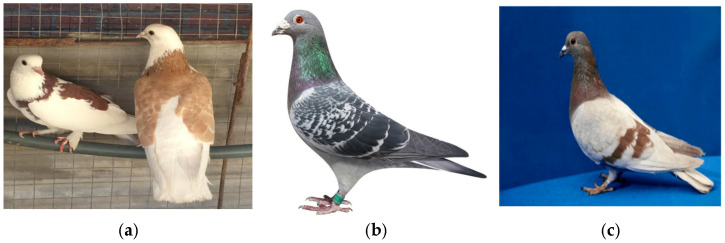
Representative individuals of the three pigeon breeds used in this study: (**a**) Tumbler pigeon; (**b**) Tarim pigeon; (**c**) homing pigeon. All individuals were healthy adults, photographed from a lateral full-body view to illustrate the typical body conformation and plumage characteristics of each breed.

This study selected Tumbler pigeons, homing pigeons, and Tarim pigeons from different regions, including Tumbler pigeons from Yanqi County (FC) and Yining County (FY), Xinjiang, China; homing pigeons from Yining City (XY), Xinjiang, China; and Tarim pigeons from Hotan City (TP), Xinjiang, China. A total of 80 blood samples were collected, with 20 healthy adult individuals per group, evenly distributed between males and females. Detailed sample information is presented in Table 1. Representative photographs of the three breeds used in this study are presented in Figure 1.

### 2.2. Sequencing Library Preparation

Total DNA was extracted from the collected blood samples using the Magnetic Bead-Based Universal Genomic DNA Extraction Kit (Catalog No.: DP705; Tiangen Biotech (Beijing) Co., Ltd., Beijing, China). After the DNA was verified to meet quality standards, it was randomly fragmented into 350 bp fragments using a Covaris ultrasonic disruptor (Covaris, Inc., Woburn, MA, USA); library construction was completed by end repair, A-tailing, adapter ligation, and PCR amplification. The constructed library was analyzed using the Agilent 5400 system (Agilent Technologies, Inc., Santa Clara, CA, USA) to assess DNA fragment integrity and insert size. Real-time quantitative PCR (qPCR) was used to determine the library’s effective concentration. Qualified libraries were sequenced at both ends on the Illumina NovaSeq X Plus platform (Illumina, Inc., San Diego, CA, USA), ultimately yielding raw sequencing data.

### 2.3. Data Quality Control Analysis

Raw sequence data (raw reads) obtained from the sequencing platform must be filtered and cleaned. The sequencing strategy is PE150 (paired-end 150 bp), with a read length of 150 bp. First, paired-end reads containing adapters are removed; when the proportion of Ns in a single-end sequencing read exceeds 10% of that read’s length, the corresponding paired-end read pair is removed; if the number of low-quality bases (Q ≤ 5) in a single-end read exceeds 50% of the read length, the corresponding paired-end read pair is removed. The average genome coverage is 14.5×. The clean reads obtained after quality control were aligned to the reference genome X101SC24104619-Z01-J001 (https://ftp.ncbi.nlm.nih.gov/genomes/all/GCF/000/337/935/GCF_000337935.1_Cliv_1.0/, accessed on 12 May 2024) using the BWA software (version 0.7.17-r1188) [14], and the alignment results were filtered to remove duplicates using SAMTOOLS software (version 1.13) [15] with the rmdup parameter. Individual SNP/Indel calling was performed using SAMTOOLS, with the requirement that SNP read support be greater than 4 and mapping quality (MQ) be greater than 20. The final SNP filtering criteria were: missing rate > 0.2, minor allele frequency (MAF) < 0.05, depth < 3. To eliminate the effects of linkage disequilibrium on downstream analyses, SNPs were pruned using the -indep-pairwise command in PLINK 1.9, with parameters set to a window size of 50 SNPs, step size of 10 SNPs, and *r*^2^ threshold of 0.2. The resulting independent SNPs were used for PCA and ADMIXTURE analyses. Finally, the obtained variant sites were annotated using ANNOVAR software (version 2015Dec14) [16].

### 2.4. Group Principal Component Analysis

Using GCTA software (version 1.24.2) [17], PCA analysis was performed on filtered single-nucleotide polymorphism (SNP) data. During analysis, loci with more than two alleles and mismatched data were excluded. This yielded a score matrix for each sample across the principal components (PCs), with the top three PCs selected for visualization.

### 2.5. Population Genetic Diversity Analysis

Using the PLINK v1.9 (https://www.cog-genomics.org/plink/, accessed on 12 May 2024) software, the number of alleles (*N*_a_), effective allele number (*N*_e_), expected heterozygosity (*H*_E_), observed heterozygosity (*H*_O_), nucleotide diversity (π), and Shannon diversity index (I) for each population were calculated; the polymorphism information content (PIC) was calculated using the Cervus v3.0s (http://www.fieldgenetics.com/pages/aboutCervus.jsp, accessed on 12 May 2024) software. All analysis parameters were set to their default values to ensure the reliability and reproducibility of the results.

### 2.6. Genetic Differentiation and Phylogenetic Tree Analysis

The genetic differentiation coefficient (*F*_ST_) between different populations was calculated using the VCFtools v0.1.16 (https://vcftools.github.io/, accessed on 12 May 2024) software; the IBS genetic distance (DR) between different populations was calculated using PLINK 1.9; the gene flow (*N*_m_) between different populations was calculated using the Arlequin software (version 3.5.2.2) (http://cmpg.unibe.ch/software/arlequin35/, accessed on 12 May 2024), and the degree of genetic differentiation and gene exchange among different populations was analyzed. The distance matrix was calculated based on the SNP results using the TreeBeST (Heng Li) software (version 1.9.2) (http://treesoft.sourceforge.net/treebest.shtml, accessed on 12 May 2024). Then, using the neighbor-joining method with bootstrap values set to 1000, the population phylogenetic tree was constructed.

### 2.7. Analysis of Population Genetic Structure

We used the Admixture software (v1.3.0) (D.H. Alexander et al., 2009) [18] to infer population genetic structure and ancestry. For the study population, we preset the number of ancestral populations (*K*) from 2 to 6 for clustering. The optimal number of clusters was determined by evaluating cross-validation (CV) error values across different *K* values, and a CV error plot was generated to illustrate the trend in CV error for each *K* value. The *K* value with the lowest CV error was selected as the optimal number of ancestral subpopulations. Based on this optimal *K* value, we visualized the Q-matrix output from Admixture using the Pophelper package in R (version 2.3.1) to generate a population-genetic structure plot.

## 3. Results

### 3.1. Sequencing Data Quality Control and Alignment

The experiment involved whole-genome resequefcing on 80 samples from four pigeon populations. As shown in Table S1, after quality control, the clean data ranged from 11.8 G to 35.03 G, with an average Q30 ratio of 95.93% and an average GC content of 43.03%. The alignment rate with the reference genome was between 95.33% and 98.32%, and the average sequencing depth was 14.5×, indicating that the sample data volume was sufficient, the sequencing quality was qualified, the GC distribution was normal, the alignment results were normal, and it could be used for subsequent variant detection and related analyses.

### 3.2. SNP Site Analysis

The number of SNP loci detected in the four pigeon populations ranged from 7,123,222 to 21,467,419 (the minimum value was from sample FY1, and the maximum value was from the combined analysis of all 80 samples). The number of InDels ranged from 685,132 to 1,794,436 (the minimum was from sample FC1 and the maximum from the combined analysis of all 80 samples). After quality control filtering, 333,680 high-quality SNPs were obtained, and the variant detection results were normal. The results of variation detection were normal. Annotations and statistics were conducted for SNPs and InDels, respectively (see Tables S2 and S3). The results show that introns account for 41.84% (8,979,960 variations), being the region where variations occur most frequently; intergenic regions account for 36.36% (7,803,866 variations), the second most frequent area for variations; exons account for 1.59% (341,206 variations); the remaining variations are distributed in regions such as Others, upstream and downstream regulatory regions, UTR, and splicing sites, totaling approximately 20.21% (including upstream, downstream, 5′UTR, 3′UTR, Splicing, and Others).

### 3.3. Group PCA Results

**Figure 2:**
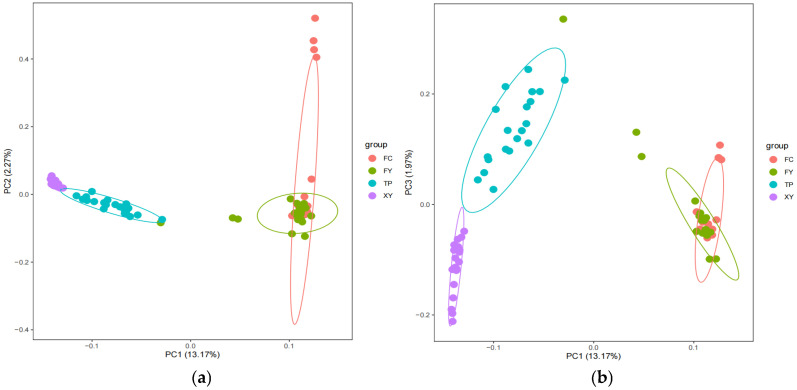
Principal component analysis (PCA) of the four pigeon populations. (**a**) PC1 versus PC2. (**b**) PC1 versus PC3.

Principal component analysis (PCA) was performed based on genome-wide SNP markers. The variance contribution rates of the first three principal components were 13.17% for PC1, 2.27% for PC2, and 1.97% for PC3, respectively. The overall variance explained was low, indicating limited genetic differentiation among the four pigeon populations, with most genetic variation residing within populations. The cumulative contribution of PC1 and PC2 was 15.44%, which effectively separated TP and XY from FY and FC, with PC2 further distinguishing TP from XY (Figure 2a). PC1 and PC3 together accounted for 15.14%, further illustrating the partial separation between FY and FC, while TP and XY each exhibited independent clustering trends (Figure 2b).

### 3.4. Analysis of Population Genetic Diversity

This study conducted a genetic diversity analysis among the FC, FY, TP, and XY populations, with results shown in Table 2. The observed number of alleles (*N*_a_) ranged from 1.93 to 1.97. In contrast, the effective number of alleles (*N*_e_) ranged from 1.55 to 1.56, with the highest (1.558) in the FY population and the lowest (1.550) in the XY population, indicating no significant overall differences. PIC values ranged from 0.251 to 0.256, all reflecting moderate polymorphism levels, suggesting that all four pigeon populations retained a moderate degree of genetic polymorphism. Observed heterozygosity (*H*_O_) and expected heterozygosity (*H*_E_) ranged from 0.496 to 0.506 and 0.314 to 0.320, respectively, with *H*_E_ values consistently lower than *H*_O_ values. Among them, the *H*_O_ values of the FY and FC populations were higher than those of TP and XY. Regarding nucleotide diversity, FY had the highest π value (0.329), while XY had the lowest (0.324). The I values for the four populations ranked as follows: FY (0.692) > TP (0.689) > FC (0.682) > XY (0.679).

### 3.5. Genetic Differentiation and Phylogenetic Tree Analysis Results

**Figure 3:**
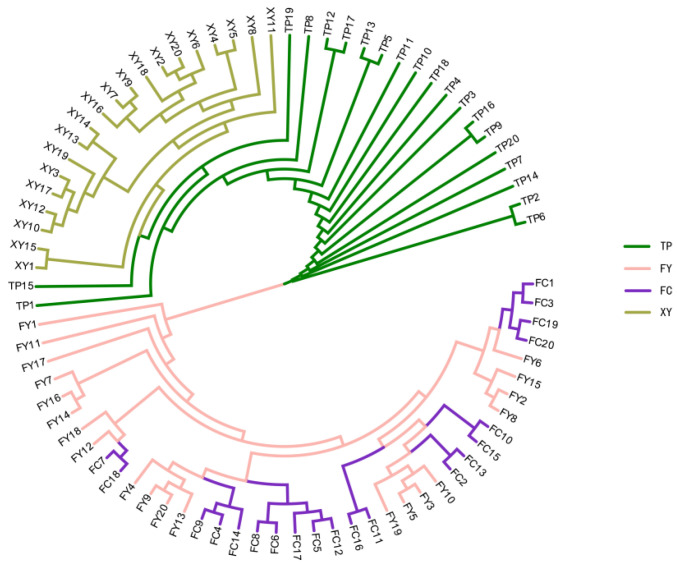
Population phylogenetic tree.

By calculating the *F*_ST_, *N*_m_, and DR values, the results in Table 3 show that the *F*_ST_ values for the four pigeon populations range from 0.009 to 0.030, all of which are <0.05. Among them, genetic exchange between the FC and FY populations is the most active (*N*_m_ = 28.93). In the constructed phylogenetic tree (Figure 3), the Tumbler pigeon populations (FC, FY) exhibited clear monophyletic clustering with the Tarim pigeon (TP) and homing pigeon (XY) populations; the TP and XY each formed independent monophyletic clades, while the Tumbler pigeon populations FC and FY from the two regions showed evidence of interbreeding. These results indicate that the degree of population genetic differentiation among the pigeon groups is extremely low, that there is some gene flow between the Tumbler pigeon groups, and that each group still retains distinguishable genetic characteristics.

### 3.6. Population Genetic Structure Analysis Results

**Figure 4:**
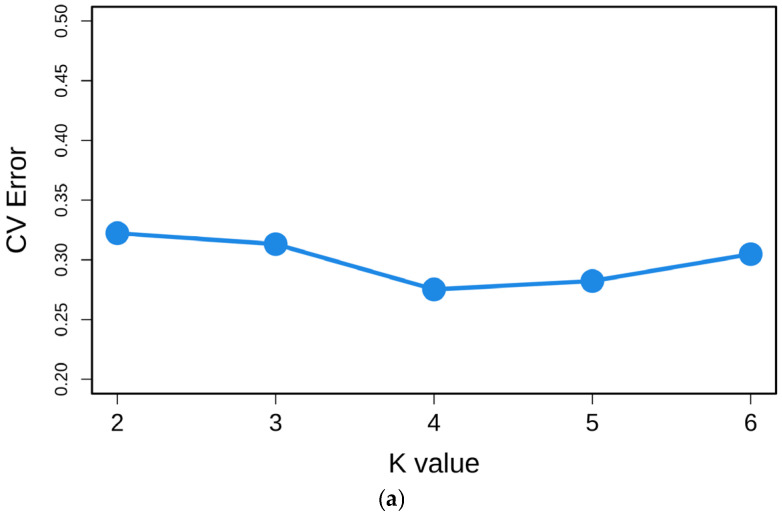

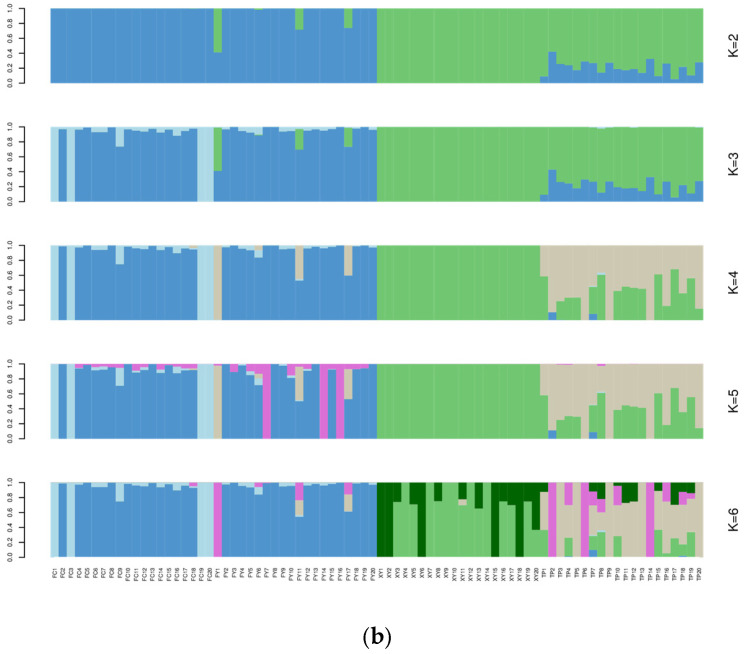
(**a**) Cross-validation (CV) error plot for different *K* values, *K* = [2, 6]; (**b**) population genetic structure plot. Each vertical bar represents an individual, and different colors (red, green, blue, yellow) represent distinct ancestral genetic components.

The average and range of membership coefficients for each *K* value in the population structure analysis are shown in Supplementary Table S4. By calculating cross-validation errors (CV error) for different *K* values [2, 6], as illustrated in Figure 4a, the lowest CV error was observed at *K* = 4 (0.26), indicating that the optimal number of ancestral populations was four. Based on the optimal *K* = 4, a population genetic structure plot was generated in Figure 4b. At *K* = 2, the four populations were divided into two major clusters: one comprising the Tumbler pigeon and the other including the messenger pigeon and Tarim pigeon, suggesting clear separation between these groups. At *K* = 3, the Tumbler pigeon group exhibited distinct genetic components and was further subdivided into two subgroups (FC and FY). At *K* = 4, the four pigeon flocks were divided into four major genetic components, and the genetic backgrounds of the samples tended to become more homogeneous; As *K* increased further, no additional or clearer differentiation in population genetic components emerged, further confirming that FC and FY populations possess independent and distinct genetic backgrounds from TP and XY populations.

## 4. Discussion

Local livestock breeds carry abundant genetic information and are valuable resources for genetic research, germplasm improvement, and the development of new varieties [19]. Population genetic structure can reveal gene flow and population differentiation. At the same time, genetic diversity reflects a population’s potential to adapt to environmental changes. This is of great significance for protecting livestock genetic resources and is also an important basis for classification [20]. Tumbler pigeons have attracted considerable attention due to their distinctive flight characteristics. However, information regarding their breed identification and phylogenetic evolution remains extremely scarce. Studies have shown that whole-genome resequencing technology has been widely applied in the analysis of population genetic structure and genetic diversity. Hou et al. [21] conducted a population genetic structure analysis of the High-Tumbler pigeon, Tarim pigeon, Dizi pigeon, Xinjiang Tumbler pigeon, and several commercial meat pigeon breeds using a 5K SNP chip. The results showed that the High-Tumbler pigeon, Tarim pigeon, Spotted pigeon, and Xinjiang Somersault pigeon clustered into a single major group, with the High-Tumbler pigeon and Tarim pigeon being closely related. Genetic diversity analysis indicated that ornamental pigeon (Spotted pigeon and Xinjiang Somersault pigeon) exhibited lower genetic diversity, while racing pigeons and meat pigeons showed relatively higher levels. Hou et al. [22] performed whole-genome resequencing on seven pigeon breeds. They found that Chinese local breeds and ornamental breeds clustered together, while all commercial breeds clustered separately, suggesting that traditional ornamental pigeon may have originated from the Tarim pigeon. ROH and LD analyses showed that ornamental pigeon had the lowest genetic diversity and the highest inbreeding, whereas the Tarim pigeon had higher genetic diversity. Xu et al. [23] used whole-genome resequencing to analyze the population genetic structure of seven meat pigeon breeds and found significant genetic admixture among certain breeds (such as Dabao, Baikanu, Hybrid King, and Tianxiang No. 1), which clustered into a single admixture group, while Silver King, Shiqi, and Shenzhen King formed independent branches. The study also found through LD decay analysis that populations with higher levels of genetic admixture exhibited greater genetic diversity. Furthermore, both PCA and phylogenetic tree analyses indicated that distinct genetic differentiation has already emerged among pigeon breeds of different utilization types.

Therefore, this study also employed whole-genome resequencing technology to analyze the genetic diversity, population genetic structure, and phylogenetic relationships of the Tumbler pigeon, using the Tarim pigeon and the homing pigeon as controls, thereby providing a theoretical basis for the conservation and utilization of the Chinese spotted dove’s genetic resources.

In the whole-genome resequencing data, the alignment rate of all samples to the reference genome exceeded 95%, with sufficient sequencing depth and a normal GC content distribution. This indicates that the selected reference genome is well-suited to the pigeon population in this study and that the data quality is reliable, meeting the requirements for downstream analysis. The number of SNP loci detected in the FY, FC, TP, and XY populations ranged from 7,123,222 to 21,467,419, while the number of InDels ranged from 685,132 to 1,794,436. These results are generally consistent with those reported in other pigeon resequencing studies [24], and the distribution patterns of variant annotations also align with expectations. The slightly higher number of InDels detected in some populations may be related to differences in sequencing depth and the accumulation of structural variations across breeds. Annotation statistics showed that the SNP variants were distributed across various genomic regions.

In population genetics research, PCA can utilize differences in single-nucleotide polymorphisms (SNPs) within individual genomes to distinguish genetic subgroups [25,26]. By combining SNP data from multiple individuals into a matrix and extracting its eigenvectors, a scatter plot can be generated to display genetic relationships among individuals visually [27,28]. In principal component analysis, PC1, PC2, and PC3 explained 13.17%, 2.27%, and 1.97% of the genetic variation, respectively. The four pigeon populations showed distinct genetic structure. The Tumbler pigeon populations as a whole were genetically distant from both the Tarim pigeon and homing pigeon populations, with the Tarim pigeon and homing pigeon each forming independent monophyletic clusters, indicating that Tumbler pigeons possess a different genetic background from Tarim pigeons and homing pigeons.

Furthermore, we observed that Tumbler pigeons from the two regions were positioned in proximity to each other, with some individuals overlapping, suggesting the possibility of a certain degree of gene flow between these two populations. The low cumulative variance contribution rate of the first three principal components in the PCA can be primarily attributed to the geographic proximity of the four pigeon populations, frequent gene flow, and limited genetic differentiation. Moreover, the vast majority of SNPs in the genome can be regarded as neutral variants [29], primarily reflecting random fluctuations in allele frequencies within individuals rather than systematic differences between populations.

Genetic diversity serves as the foundation for the survival and continuous evolution of organisms in their environment, reflecting their ability to survive and evolve in different conditions [30,31]. Balog et al. [32] conducted a genetic diversity analysis of domestic pigeon breeds from Central Europe (the Carpathian Basin) and the Middle East (Iraq) using mitochondrial COI sequences. They found significant differences in genetic diversity among pigeon populations of different geographic origins, with breeds from the Carpathian Basin exhibiting higher diversity, while those from Iraq showed lower diversity. The analysis indicated that pigeon breeds from the Carpathian Basin are more closely related to Asian breeds than to Mediterranean breeds, suggesting that this region may have served as a geographical bridge between Asia and Europe during the dispersal of pigeon breeds. *N*_e_, *H*_E_, and PIC can all be used to estimate the degree of genetic variation within a population and to determine the distribution of genetic diversity [33]. Among them, *N*_e_ refers to the ideal population size that has the same variance in gene frequencies or the same rate of heterozygosity attenuation as the actual population; *H*_E_ is the core indicator of genetic diversity within a population, reflecting the theoretical degree of allelic heterozygosity. The results of this study indicate that there are no significant differences in *N*_a_ and *N*_e_ among the FC, FY, TP, and XY groups; the PIC values for the four populations indicate that all populations retain moderate genetic polymorphism. Notably, the *H*_O_ values for all four pigeon populations were higher than their *H*_E_ values. The *H*_O_ values for the FY and FC populations were higher than those for the TP and XY populations, suggesting that the Tumbler pigeon population has relatively rich genetic diversity and a lower risk of inbreeding. This discrepancy may result from two factors: first, the small sample size in each population (*n* = 20), under which *H*_E_ is prone to underestimation; second, as local Xinjiang breeds, the Tumbler, homing, and Tarim pigeon have long been subject to a semi-open breeding system where artificial selection coexists with natural selection, potentially resulting in the retention of high true heterozygosity within the populations. This study employed strict genotyping standards for SNP detection and LD pruning to eliminate potential linkage disequilibrium. The Wahlund effect typically leads to heterozygote loss, which is contrary to the observations in this study; therefore, it can be ruled out as a major factor. Both the π and I values for the FY population were higher than those for the TP and XY populations, indicating that the Tumbler pigeon population exhibits higher genetic diversity, which may be related to its unique breeding history and population structure.

Podbielska et al. [34] analyzed the population genetic structure of Polish racing pigeons using microsatellite markers. The results showed that the population genetic structure of racing pigeon was heterogeneous, with no clear differentiation by place of origin, suggesting high gene flow among regions. The study also found that the genetic diversity of the racing pigeon population was at a relatively satisfactory level, with the inbreeding coefficient not reaching the warning threshold. This suggests that, despite certain selection pressures, genetic diversity has been well maintained. We employed three genetic distance measures—*F*_ST_, *N*_m_, and IBS—to investigate genetic differentiation and kinship among the four pigeon populations. We used three genetic distance indicators, namely *F*_ST_, *N*_m_, and IBS, to assess the genetic differentiation and phylogenetic relationship among the four pigeon populations. Among them, *F*_ST_ measures the degree of genetic differentiation between subgroups, and a higher *F*_ST_ value indicates greater differentiation [35,36]. *N*_m_ refers to the process by which individuals disperse from their origin, leading to gene exchange between populations, which can occur within the same or different species. The results showed that *F*_ST_ values among the four populations ranged from 0.009 to 0.030, indicating low genetic differentiation. Among them, the genetic distance between FC and XY was the greatest. The two populations were the most distantly related, suggesting that long-term artificial selection or geographic isolation may have led to the formation of relatively independent genetic backgrounds in these two groups; Conversely, the *F*_ST_ value between FC and FY was the lowest, indicating extremely weak genetic differentiation and the highest level of gene flow, suggesting that genetic exchange between these two Tumbler pigeon populations is relatively active. Although the overall level of genetic differentiation among the groups is low (*F*_ST_ < 0.05), we must remain vigilant against increasing genetic isolation among subpopulations. It is recommended to manage mating between unrelated individuals, monitor inbreeding, and control the exchange of regional Tumbler pigeon populations.

The phylogenetic evolutionary tree is an analytical method in bioinformatics used to describe the relationships among organisms or genes. It can utilize the tree-like bifurcation pattern to explain the genetic relationships between organisms or genes from an evolutionary perspective [37], and thus is a powerful tool for determining the degree of biological evolution and genetic relationships [38]. This study revealed distinct evolutionary characteristics among the Tumbler pigeon (FC, FY) and TP and XY populations, with each forming independent monophyletic branches and showing genetic independence. This indicates that Tumbler pigeon, Tarim pigeon, and homing pigeon each possess unique genetic backgrounds. However, individual crossover was observed between Tumbler pigeon from the two regions, suggesting that no significant genetic barrier has formed between the FC and FY populations during their evolutionary process. Studies have shown that hybridization between domestic pigeon and their ancestral populations can lead to genetic homogenization.

In contrast, extensive hybridization among different breeds and gene flow are major reasons for the low level of genetic differentiation among domestic pigeon breeds [39]. Based on this, it is speculated that interbreeding between the FC and FY populations may be related to human-mediated gene flow facilitated by the annual racing pigeon competitions and auctions held between the two regions; however, this hypothesis requires further verification through detailed research. By combining principal component analysis, genetic diversity analysis, and phylogenetic tree analysis, it can be shown that there is significant genetic background differentiation among the populations of the Tumbler pigeon compared to the Tarim pigeon and the homing pigeon, suggesting that the genetic background of the Tumbler pigeon differs significantly from that of the other two pigeon breeds.

Population genetic structure infers the proportions of ancestral components of individuals from genotype data, revealing genetic stratification and the degree of admixture within the population. This study found that when the ancestral population size was low (*K* = 2), the four pigeon groups could be divided into two major genetic lineages, with clear clustering among the Tumbler pigeon, the homing pigeon, and the Tarim pigeon. When *K* = 4, the four main genetic groups within the pigeon population were identified, yielding the best clustering. As ancestral population size increased, each pigeon group was clearly distinguished from the others. Cross-penetration occurred among the Tumbler pigeon groups, which may be related to genetic exchanges between the ancestors of Tumbler pigeon in the two regions. This further indicates that the Tumbler pigeon groups, the Tarim pigeon group, and the homing pigeon group each have independent and distinct genetic backgrounds.

This study investigated the genetic diversity, genetic structure, and phylogenetic relationships of three pigeon populations from different regions (the Tumbler pigeon, the homing pigeon, and the Tarim pigeon). It clarified the unique genetic status of the Tumbler pigeon and the patterns of differentiation among the populations, providing important genomic evidence for the conservation and utilization of pigeon genetic resources. It should be noted that the sample size of 20 individuals per group in this study is suitable for exploratory genomic surveys; however, the statistical power remains relatively limited when inferring genetic distinctiveness at the breed level, and the study covers only these three pigeon populations. Therefore, the genetic status assessment of the Tumbler pigeon in this study remains preliminary. Subsequent studies should validate these findings by increasing sample size and incorporating functional analyses. In the future, more local pigeon breeds and wild pigeon ancestors could be included to refine the evolutionary landscape of pigeon further.

Furthermore, the genetic basis for phenotypic differences among these populations remains to be thoroughly explored. Future studies will combine methods such as linkage disequilibrium decay, homozygous blocks, and selective scanning analyses to identify candidate genes associated with the phenotypic differences among the aforementioned populations, thereby further elucidating the molecular mechanisms underlying the formation of specific phenotypes in populations such as the Tumbler pigeon. The findings of this study provide theoretical support for the conservation and genetic improvement of local pigeon breeds, and offer methodological references for population genetics research on other livestock and poultry species.

## 5. Conclusions

Using whole-genome resequencing, this study systematically analyzed the genetic diversity, genetic structure, and phylogenetic relationships of the Tumbler pigeon, the Tarim pigeon, and the homing pigeon. The results indicate that the Tumbler pigeon exhibits a moderate level of genetic diversity and shows detectable but low genetic differentiation from the Tarim pigeon and homing pigeon populations. This reveals distinct differences in population genetic structure among the three pigeon populations in the Xinjiang region, suggesting that the Tumbler pigeon has potential for conservation and use as a distinctive local genetic resource. These findings, at the genomic level, support the uniqueness and value of the Tumbler pigeon as a distinctive local genetic resource. They provide a molecular basis for its germplasm characterization and priority conservation, which is of great importance for maintaining the genetic diversity of local pigeon breeds in Xinjiang. The constructed population genetic framework also provides a data foundation for subsequent systematic evaluations of pigeon germplasm resources and scientific assessments of conservation priorities for local breeds.

## Figures and Tables

**Table 1:** Sample collection information.

Group	Sample ID Range	Sample Size (♂/♀)	Geographic Origin
Tumbler pigeon (FC)	FC1–FC20	10/10	Yanqi, Xinjiang, China
Tumbler pigeon (FY)	FY1–FY20	10/10	Yining, Xinjiang, China
Homing pigeon (XY)	XY1–XY20	10/10	Yining, Xinjiang, China
Tarim pigeon (TP)	TP1–TP20	10/10	Hotan, Xinjiang, China

**Table 2:** Genetic diversity statistics of the four pigeon populations.

Group	*N*_a_	*N*_e_	PIC	*H*_O_	*H*_E_	π	I
FC	1.940	1.553	0.251	0.503	0.316	0.325	0.682
FY	1.953	1.558	0.256	0.506	0.320	0.329	0.692
TP	1.969	1.548	0.253	0.496	0.317	0.326	0.689
XY	1.928	1.550	0.251	0.497	0.314	0.324	0.679

**Table 3:** Statistical analysis of genetic differentiation among 4 pigeon populations.

Group 1	Group 2	*F*_ST_	*N*_m_	DR
FC	FY	0.009	28.93	0.006
FC	TP	0.020	11.79	0.016
FC	XY	0.030	8.17	0.025
FY	TP	0.019	12.84	0.015
FY	XY	0.028	8.63	0.023
TP	XY	0.012	20.57	0.008

## Data Availability

The raw sequencing data generated in this study will be deposited in the NCBI Sequence Read Archive (SRA). The accession number will be provided upon manuscript acceptance. Other original contributions presented in this study are included in the article and Supplementary Materials. Further inquiries can be directed to the corresponding authors.

## Supplementary Materials

The following supporting information can be downloaded at https://www.mdpi.com/article/10.3390/ani16152343/s1: calculation formula; Supplementary File S1. Na_Ho_Statistics; Table S1. Sequencing data control and alignment. Table S2. InDel Detection and Annotation Result Statistics. Table S3. SNP Detection and Annotation Result Statistics. Table S4. Q-matrix results for different *K* values.

## Abbreviations

The following abbreviations are used in this manuscript:

TP	Tarim pigeon
XY	Homing pigeon
FC, FY	Tumbler pigeon
*N*_a_	The number of alleles
*N*_e_	The number of effective alleles
PIC	Polymorphism information content
*H*_O_	Heterozygosity
*H*_E_	Heterozygosity
WGS	Whole-genome resequencing
Raw reads	Raw sequence data
SNP	Single-nucleotide polymorphism
PCs	Principal components
π	Nucleotide diversity
I	Shannon diversity index
*F*_ST_	Genetic differentiation coefficient
DR	Genetic distance
*N*_m_	Number of migrants per generation
CV	Cross-validation
PCA	Principal component analysis
