# Supplementary material for: Population Structure and Genetic Diversity of Tumbler Pigeon Based on Whole-Genome Resequencing
Source: Animals (Basel). 2026 Aug 1;16(15):2343. doi: 10.3390/ani16152343 (PMC13463516; doi:10.3390/ani16152343)
Supplement: Supplementary file 1 [file animals-16-02343-s001.zip › calculation formula.pdf]

1、Genetic Diversity Statistics

1.1 Effective Group Calculation (Ne)

Effective population size (Ne) is a key concept in population genetics that describes the size of an idealized population exhibiting identical genetic drift and inbreeding levels to the actual population.

Linkage disequilibrium method: Estimates Ne by observing linkage disequilibrium between alleles.

$$N_e = \frac{1}{4c \cdot r^2}$$

Here, cc denotes the linkage interval, and r^2 represents the linkage disequilibrium coefficient.。

1.2 Calculation of average number of alleles (Na)

The average number of alleles (Na) refers to the abundance of alleles, representing the mean number of alleles at a specific genetic locus. It serves as a critical indicator of a population's capacity to maintain allele diversity; a high average effective number of alleles indicates that the population is less susceptible to disruption and subsequent emergence of new variants under genetic drift pressure. The calculation formula is as follows::

$$\mathrm{Na} = \frac{\sum_{i=1}^{n} Na_i}{N}$$

In the formula:

Nai refers to the number of alleles at the i-th locus:

N – Total number of measurement points。

1.3 Calculation of Polymorphic Information Content (PIC)

Polymorphic information content (PIC) measures the probability that a offspring acquires a specific genetic marker from its father's or mother's identical allele, serving as an indicator of DNA variation levels. Proposed by Botstein in 1980 for assessing population polymorphism, the PIC value of a marker is calculated based on its allele frequency using the following formula:

$$\mathrm{PIC} = 1 - \sum_{i=1}^{n} P_i^2 - \sum_{i=1}^{n-1} \sum_{j=i+1}^{n} 2P_i^2 P_j^2$$

In the formula:

n – Number of alleles; Pi – Frequency of the i-th allele in the population; Pj – Frequency of the j-th allele in the population; PIC – The arithmetic mean of all PIC values across all loci. A PIC value> 0.5 indicates high polymorphism, 0.25 <PIC <0.5 indicates moderate polymorphism, and PIC <0.25 indicates low polymorphism.

1.4 Genetic differentiation coefficient (Fst)

The interpopulation genetic differentiation index (Fst) is a measure of population divergence and genetic distance; a higher Fst value indicates greater genetic difference. It is suitable for comparing diversity among subpopulations and serves to quantify the degree of population divergence, with values ranging from 0 to 1. A Fst value of 0 signifies random mating between populations and complete genotype similarity; a value of 1 indicates complete isolation and no genetic similarity. The Fst calculation formula is as follows:

$$Fst = \frac{Ht - Hs}{Ht}$$

In the formula:

Ht: The expected heterozygote frequency of the entire population relative to the ideal population; HS: The average expected heterozygote frequency of a local population relative to the ideal population.

FST: The ratio of the reduction in HS to HT, representing the average inbreeding coefficient among populations with genetic proximity.

The calculation of group Fst is the arithmetic mean of Fst values across all loci.

Additionally, there are:

$$F_{IS} = \frac{H_S - H_I}{H_S}$$

$$F_{IT} = \frac{H_T - H_I}{H_T}$$

HI: Average frequency of heterozygotes observed across the entire population FIS: Average inbreeding coefficient of the local population FIT: Average inbreeding coefficient of the entire population

1.5 Nucleotide diversity (Pi)

In population genetics, the π (Pi) value is used to measure the degree of genetic diversity within a population. A higher value indicates greater nucleotide diversity, while a lower value signifies less diversity. The calculation formula for Pi is as follows:

$$\pi = \sum_{j=1}^{S} \frac{N(1-\sum_{i=1}^{n} p_i^2)}{N-1}$$

$$\theta\pi = \frac{\pi}{S}$$

In the formula:

S － Number of separation sites; N － Number of sequences in the sample (e.g., for 10 samples in a population, the number of diploid sequences is N = 20); n － Number of alleles; Pi － Frequency of the i-th allele.

1.6 Nei's standard genetic distance (DR)

Genetic distance (DR) refers to the degree of genetic variation between different populations, with the commonly used measure being the standard genetic distance proposed by Nei (1972), calculated as follows:

$$J_x = \sum_{i=1}^{n}\sum_{j=1}^{k}\frac{X_{ij}^2}{n},\ J_y = \sum_{i=1}^{n}\sum_{j=1}^{k}\frac{Y_{ij}^2}{n}, J_{yx} = \sum_{i=1}^{n}\sum_{j=1}^{k}\frac{X_{ij}Y_{ij}}{n}$$

$$D_s = \text{-}Ln\frac{J_{xy}}{\sqrt{J_x J_y}}$$

1.7 Hardy-Winberg equilibrium test (HWB test)

The Hardy-Weinberg law states that, under ideal conditions, the frequencies of alleles remain stable and unchanged during inheritance, maintaining genetic equilibrium.

Calculate the expected counts of genotypes AA, Aa and aa based on Hardy-Weinberg Balance

EnAA=PA2

NEnAa=2PAPa

NEnaa=Pa2N

χ2 test:

χ2=∑En(En−n)2

df=2. According to the χ2 distribution table, if χ2>5.99, then P<0.05, indicating that the gene frequencies deviate from the Hardy-Weinberg equilibrium law.

Where: En− Expected number of individuals of each genotype in the population

n− Observed number of individuals of each genotype in the population

N− Total number of individuals in the population

PA,Pa− Allele frequencies in the population

1.8 gene flow Nm

Gene flow (Nm): refers to the process of genetic exchange between populations resulting from the dispersal of individuals from their original habitats, occurring among populations of the same or different species. The primary function of gene flow is to reduce genetic differences between populations. In the island model, Nm represents the number of effective migrants per generation, i.e., an estimated value of gene flow: Nm = 1/4(1/Fst − 1).

$$Nm = \frac{1}{4}(\frac{1}{Fst} - 1)$$

FST: The ratio of the reduction in HS to HT, representing the average inbreeding coefficient among populations with genetic proximity.

Nm: Calculation of Nm for gene flow populations: the arithmetic mean of Nm values across all loci

1.9Shannon Diversity Index (abbreviated as I)

The Shannon-Wiener index employs methods from information theory; a higher value indicates greater community diversity and consequently greater uncertainty.

I=－∑iSpi∗ln(pi)

I: Shannon Polymorphism Index
S: Total number of varieties (genotypes in this study)
pi: Proportion of variety (genotype) i in the total population
ln: Natural logarithm with base constant e, often replaced by log2 (log2 is adopted in this analysis)

Calculation of Group I: the arithmetic mean of all site I values
